# Homozygous KSR1 deletion attenuates morbidity but does not prevent tumor development in a mouse model of RAS-driven pancreatic cancer

**DOI:** 10.1371/journal.pone.0194998

**Published:** 2018-03-29

**Authors:** Elizabeth A. Germino, Joseph P. Miller, Lauri Diehl, Carter J. Swanson, Steffen Durinck, Zora Modrusan, Jeffrey H. Miner, Andrey S. Shaw

**Affiliations:** 1 Department of Pathology and Immunology, Washington University School of Medicine, St. Louis, Missouri, United States of America; 2 Department of Research Biology, Genentech, South San Francisco, California, United States of America; 3 Division of Nephrology, Washington University School of Medicine, St. Louis, Missouri, United States of America; 4 Department of Pathology, Genentech, South San Francisco, California, United States of America; 5 Department of Bioinformatics and Computational Biology, Genentech, South San Francisco, California, United States of America; 6 Department of Molecular Biology, Genentech, South San Francisco, California, United States of America; Indiana University School of Medicine, UNITED STATES

## Abstract

Given the frequency with which MAP kinase signaling is dysregulated in cancer, much effort has been focused on inhibiting RAS signaling for therapeutic benefit. KSR1, a pseudokinase that interacts with RAF, is a potential target; it was originally cloned in screens for suppressors of constitutively active RAS, and its deletion prevents RAS-mediated transformation of mouse embryonic fibroblasts. In this work, we used a genetically engineered mouse model of pancreatic cancer to assess whether KSR1 deletion would influence tumor development in the setting of oncogenic RAS. We found that *Ksr1*^*-/-*^ mice on this background had a modest but significant improvement in all-cause morbidity compared to *Ksr1*^*+/+*^ and *Ksr1*^*+/-*^ cohorts. *Ksr1*^*-/-*^ mice, however, still developed tumors, and precursor pancreatic intraepithelial neoplastic (PanIN) lesions were detected within a similar timeframe compared to *Ksr1*^*+/+*^ mice. No significant differences in pERK expression or in proliferation were noted. RNA sequencing also did not reveal any unique genetic signature in *Ksr1*^*-/-*^ tumors. Further studies will be needed to determine whether and in what settings KSR inhibition may be clinically useful.

## Introduction

KSR was originally cloned in *Drosophila* and *C*. *elegans* in separate screens for loss of function mutations that suppressed a constitutively active RAS protein [1–3]. KSR shares high homology with the RAF family of kinases, but the mammalian form lacks a catalytic lysine and so it has been characterized as a pseudokinase. It is proposed to function as a scaffold for RAF, MEK and ERK [3–9]. There are two isoforms, KSR1 and KSR2; data compiled in the Human Protein Atlas indicates that KSR1 mRNA expression is more ubiquitous, while KSR2 is mainly expressed in the brain [10]. *Ksr1*^*-/-*^ mice are phenotypically normal but have modest defects in T cell activation and long-term associative memory formation [11, 12]. In a mouse model of breast cancer based on a viral oncogene, the absence of KSR1 results in reduced tumor frequency and progression [11].

Mouse embryonic fibroblasts derived from *Ksr1*^*-/-*^ mice exhibit a significant reduction in EGF- and TPA-induced MAPK activation [13] and importantly, KSR1 is required for RAS-induced transformation [14, 15]. Furthermore, in a v-Ha-ras mouse model of skin papilloma, KSR1 deletion attenuates papilloma formation [13]. Lastly, antisense oligonucleotides to KSR1 are able to reduce tumor growth of KRAS-dependent human pancreatic carcinoma xenografts in nude mice [16].

These data support a role for KSR1 in the context of RAS-driven tumor growth, but KSR1 deficiency has not yet been tested in a genetically engineered mouse model of constitutively active RAS. One well-studied mouse model of RAS-driven pancreatic cancer combines a conditional oncogenic KRAS allele induced by a pancreatic-specific Cre on the background of a loss of either the tumor suppressor p53 or INK4/ARF, (reviewed in [17, 18]). This model recapitulates the progression of histological lesions that characterize human pancreatic ductal adenocarcinoma [19]. In this work, we sought to establish whether KSR1 deletion could influence RAS-driven tumorigenesis *in vivo* using this mouse model of pancreatic ductal adenocarcinoma (PDAC).

## Methods

### Mice

Mouse strains used in this study were: *Ksr1*^*-/-*^ [11], *Pdx1-Cre* [19], *p53*^*flox/flox*^ [20], *LSL*-*Kras*^*G12D/*+^ [21]. Mice were maintained on a C57BL/6 background. All mouse experiments were conducted with prior approval of the Washington University Animal Care and Use Committee. Animals were kept in conventional animal facilities and monitored daily by trained husbandry staff. Weights were charted weekly. Criteria for a humane endpoint were chosen to minimize animal suffering and included any weight loss or gain outside expected physiologic changes, changes in physical appearance that could cause distress including the development of rectal prolapse or other anatomic abnormalities, and changes in behavior including signs of lethargy. When required, mice were euthanized with CO2 according to Institutional Animal Care and Use Committee (IACUC) guidelines. Consistent with our intent to maintain a humane endpoint, tumor burden in sacrificed mice was always found to be less than 20mm. Although rare, mice that died before showing any signs of morbidity were analyzed with necropsy when possible. In these cases, tumor burden was never found to be more than 20mm.

### Histology

Immediately after animal sacrifice, the whole pancreas plus any visible tumor was removed for histological analysis. To study the phenomenon of rectal prolapse, the gastrointestinal tract was removed from the duodenum to the rectum. Immunohistochemistry (IHC) was performed on formalin-fixed paraffin-embedded tissue sections mounted on glass slides. Multiple slides from each block were generated to reduce sampling error. All IHC steps were carried out on the Ventana Discovery XT (Ventana Medical Systems) autostainer. Stained slides were independently reviewed in blinded fashion by a pathologist (L.D.) to assess pattern and strength of staining. Primary antibodies: phospho-p44/42 MAPK (ERK1/2; Thr202/Tyr204, Cell Signaling Technology) and Ki67 (clone SP6, LabVision/Thermo Fisher) were used at the concentration of 1μg/mL and a 1:200 dilution, respectively. Phospho-p44/42 MAPK was incubated on slides for 60 minutes at room temperature. Ki67 was incubated on slides for 32 minutes at 37°C. Ventana OmniMap anti-Rabbit HRP was used as the detection system.

### Next-generation sequencing and RT-qPCR

Snap-frozen tumor samples were processed using the AllPrep DNA/RNA isolation kit (Qiagen) according to the manufacturer’s protocol to extract both DNA and RNA. Tissue homogenization and lysis was performed with the TissueLyser II (Qiagen). Approximately 30mg of tissue sample was added to 600μL RLT buffer with a sterilized stainless steel bead (3mm diameter) in a 2mL safe-lock microcentrifuge tube, then quickly transferred to the TissueLyser for 2 minutes at 25 Hz. Lysate was centrifuged at maximum speed for 3 minutes. Supernatant was transferred to an AllPrep DNA spin column before proceeding to the AllPrep protocol. Clean-up for RNA samples was done using the RNEasy MinElute kit (Qiagen) according to the manufacturer’s protocol.

Gene expression profiling with RNA-seq was done using a total of 19 tumor samples with the following genotypes: 9 *Ksr1*^*-/-*^ tumors, 7 *Ksr1*^*+/-*^ tumors and 3 *Ksr1*^*+/+*^ tumors. Quality control of samples was done to determine RNA quantity and quality prior to their processing by RNA-seq. The concentration of RNA samples was determined using NanoDrop 8000 (Thermo Fisher Scientific) and the integrity of RNA was determined by Fragment Analyzer (Advanced Analytical Technologies). 0.5μg of total RNA was used as an input material for library preparation using TruSeq RNA Sample Preparation Kit v2 (Illumina). Size of the libraries was confirmed using 4200 TapeStation and High Sensitivity D1K screen tape (Agilent Technologies) and their concentration was determined by qPCR based method using Library quantification kit (KAPA). The libraries were multiplexed and then sequenced on HiSeq4000 (Illumina) to generate ~30 million single end 50 base pair reads.

RNASeq reads were aligned to the human genome version GRCh38 using GSNAP. Expression counts per gene were obtained by counting the number of reads aligned concordantly within a pair and uniquely to each gene locus as defined by NCBI and Ensembl gene annotations and RefSeq mRNA sequences. Differential gene expression analysis was performed using edgeR.

RT-qPCR was performed on RNA extracted from 13 of the 19 tumor samples analyzed for RNASeq. RNA was reverse transcribed using Superscript III First-Strand Synthesis System for RT-PCR (Thermo Fisher Scientific) with a starting concentration of 500 ng of RNA and using Oligo(dT) primers. cDNA from colon, lung and ovary of C57BL/6 mice (Zyagen) was used as a positive control for KSR2 expression. qPCR was performed using Taqman Universal PCR Master Mix (Thermo Fisher Scientific) and Taqman primers Mm02745105_m1 (KSR2) and Mm01171435_gH (EEF2) with FAM-MGB dyes (Thermo Fisher Scientific) with 800ng starting cDNA. qPCR was performed on a Quant Studio 7 Flex (Thermo Fisher Scientific) using the ΔΔCt method. All samples were run with 3 technical replicates on a single MicroAmp Optical 96-well Reaction Plate covered with Optical Adhesive Film (Thermo Fisher Scientific).

### Statistical analysis

Prism (version 7; GraphPad) was used for analysis and graphing of survival cohorts and qPCR data. Kaplan-Meier survival statistics were calculated using the log-rank test. P values < 0.05 were considered statistically significant. For qPCR data, comparisons of mean ΔCt for the gene of interest relative to a reference gene were made for each genotype, with individual tumor samples treated as biological replicates, using one-way ANOVA with Sidak correction for multiple comparisons.

## Results

### *Ksr1*^*-/-*^ mice on the p53 flox/wt background have a slight decrease in all-cause morbidity

*Ksr1*^*-/-*^ mice [11] were bred to the previously described PDAC model with *Pdx1-Cre*, *LSL*-*Kras*^*G12D/*+^ and *Trp53*^*flox/flox*^ [22] to generate mice that were homozygous and heterozygous for KSR1 on the background of p53 homozygous deletion and heterozygous expression of KRAS G12D. Since the *Ksr1* and *p53* genes are both on chromosome 11, about 3mB apart, we initially bred and identified recombinants between these two loci. Haemotoxylin and eosin staining of the pancreas of *Pdx1-Cre*;*LSL*-*Kras*^*G12D/*+^*;Trp53*^*flox/flox*^*;Ksr1*^*-/-*^ mice sacrificed at several time points confirmed the presence of pancreatic ductal adenocarcinoma with tumors identified in all mice between 3–4 weeks of age. We suspected that the rapid onset and aggressiveness of tumors in this model might obscure more subtle differences between knockout and wild-type mice. We therefore generated animals with p53 heterozygosity instead of p53 homozygous deletion.

**Fig 1:**
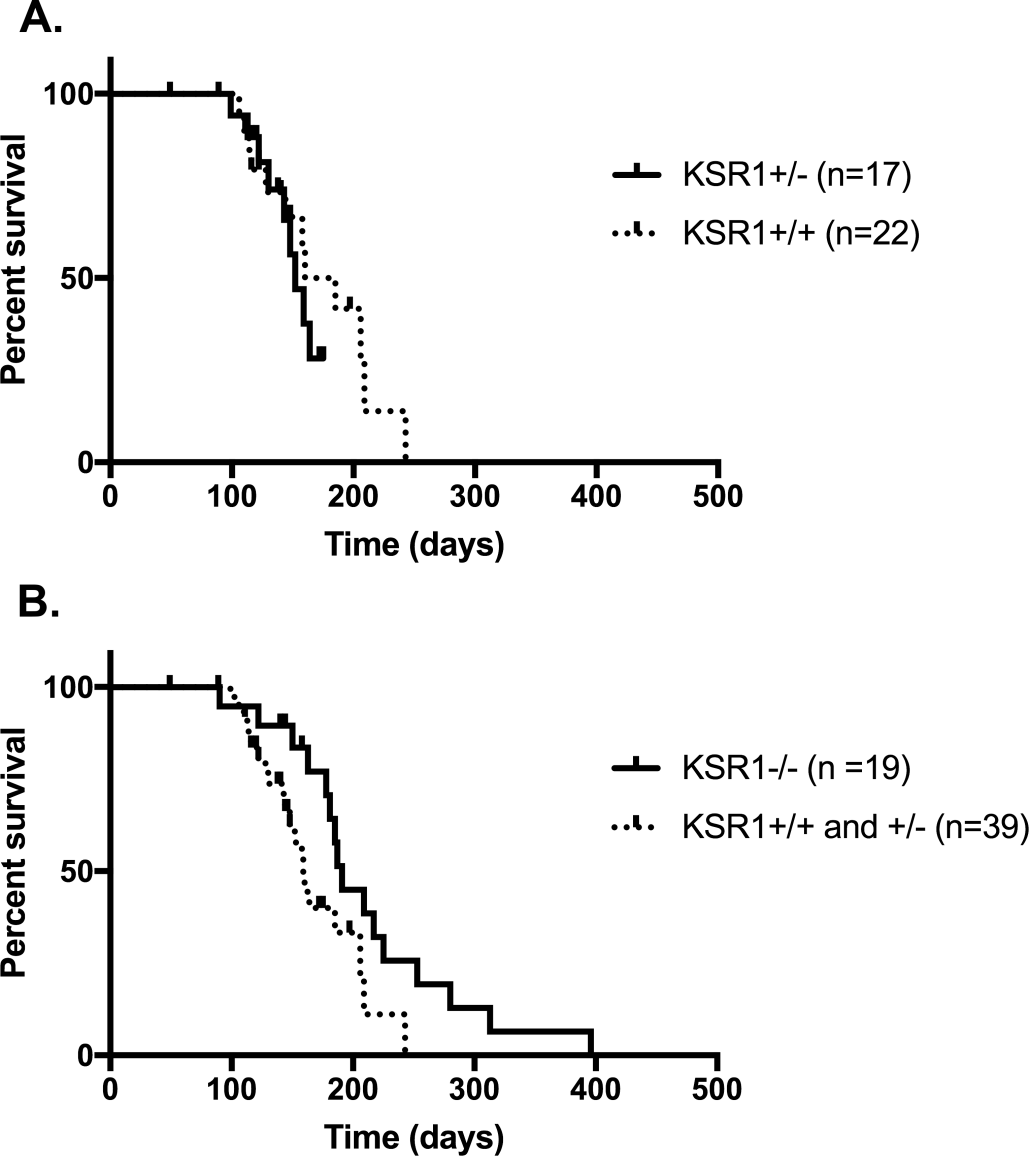
*Pdx1-Cre*;*LSL*-*Kras*^*G12D/*+^*;Trp53*^*flox/wt*^*;Ksr1*^*-/-*^ mice have a modest but statistically significant decrease in all-cause morbidity. A. Kaplan-Meier curves for *Pdx1-Cre*;*LSL*-*Kras*^*G12D/*+^*;Trp53*^*flox/wt*^*;Ksr1*^*+/+*^ and *Ksr1*^*+/-*^ mice based on age at sacrifice or death. 8 *Ksr1*^*+/-*^ and 10 *Ksr1*^*+/+*^ mice had to be censored. Median age at sacrifice or death was 152 days for *Ksr1*^*+/-*^ mice and 160 days for *Ksr1*^*+/+*^mice; there was no statistically significant difference between the two groups (p = 0.4683 by log-rank test). B. Kaplan-Meier curves for *Pdx1-Cre*;*LSL*-*Kras*^*G12D/*+^*;Trp53*^*flox/wt*^*;Ksr1*^*-/-*^, and *Ksr1*^*+/-*^ combined with *Ksr1*^*+/+*^ mice based on age at sacrifice or death. 3 *Ksr1*^*-/-*^ were censored. There was a modest but statistically significant difference between median age at sacrifice or death for *Ksr1*^*-/-*^ mice and the control group (191 and 159 days, p = 0.0344 by log-rank test).

We generated *Ksr1*^*-/-*^, *Ksr1*^*+/-*^, and *Ksr1*^*+/+*^ cohorts on the *Pdx1-Cre*;*LSL*-*Kras*^*G12D/*+^*;Trp53*^*flox/wt*^ background; unless otherwise stated, we use *Ksr1*^*-/-*^, *Ksr1*^*+/-*^, or *Ksr1*^*+/+*^ as shorthand to refer to these cohorts which always have the background genotype *Pdx1-Cre*;*LSL*-*Kras*^*G12D/*+^*;Trp53*^*flox/wt*^. Mice were followed weekly and sacrificed after changes in weight and/or signs of lethargy. Due to ethical concerns, mice were also sacrificed if they developed any other abnormality that might cause unnecessary suffering, including rectal prolapse. Ages of mice at sacrifice or death were plotted in a Kaplan-Meier survival curve (Fig 1). The difference in survival curves for *Ksr1*^*+/-*^ and *Ksr1*^*+/+*^ mice was not statistically significant (Fig 1A), and so they were grouped together to create one control group. When *Ksr1*^*-/-*^ mice were compared with *Ksr1*^*+/-*^ and *Ksr1*^*+/+*^ controls (Fig 1B), there was a modest but statistically significant decrease in all-cause morbidity (median age at sacrifice or death of 191 and 159 days respectively, p = 0.0344 by log-rank comparison). When segregated by gender, it was noted that the 4 of the 7 *Ksr1*^*-/-*^ mice sacrificed at 200 days or older mice were female, driving the overall *Ksr1*^*-/-*^ phenotype (S1 Fig). However, despite this trend the sample size was too small to detect a statistically significant difference between male and female mice. A more complete description of mice in these cohorts is included in S1 Table.

### *Ksr1*^*-/-*^ mice develop PanIN lesions by 3 months of age

**Fig 2:**
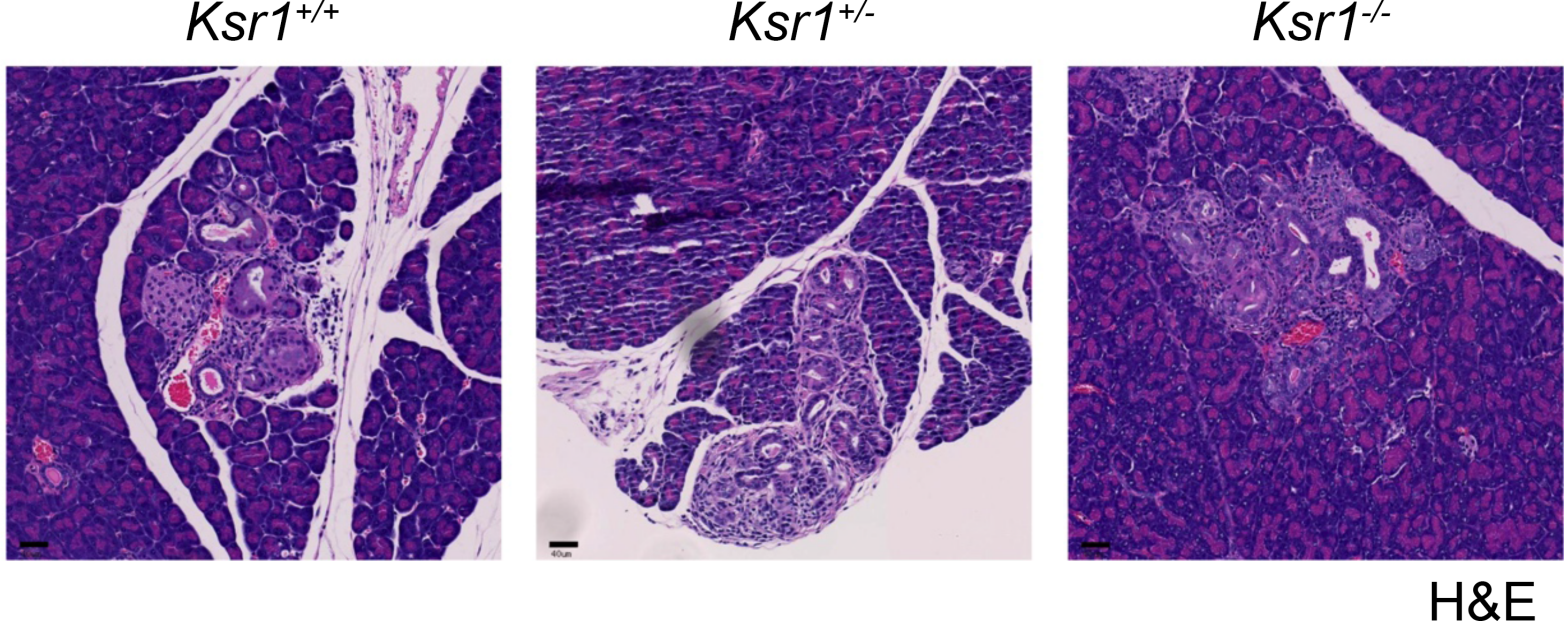
*Pdx1-Cre;LSL-Kras*^*G12D/+*^*;Trp53*^*flox/wt*^*;Ksr1*^*-/-*^ mice develop PanIN lesions at similar rates and severity to *Ksr1*^*+/-*^ and *Ksr1*^*+/+*^ mice. H&E staining of pancreatic tissues from *Pdx1-Cre*;*LSL*-*Kras*^*G12D/*+^*;Trp53*^*flox/wt*^*;Ksr1*^*+/+*^, *Ksr1*^*+/ -*^, and *Ksr1*^*-/-*^ mice sacrificed at 3 months of age highlights PanIN lesions surrounded by normal tissue (bar = 40μm).

Because *Ksr1*^*-/-*^ mice had a modest improvement in overall morbidity, we investigated whether there might be a delay in the appearance of pre-cancerous pancreatic intraepithelial neoplasms (PanIN). We sacrificed a cohort of mice from each genotype (3 *Ksr1*^*+/+*^, 3 *Ksr1*^*+/-*^, and 9 *Ksr1*^*-/-*^) at 12 weeks of age and prepared the pancreas for histological analysis. We observed characteristic noninvasive PanIN lesions, comprising columnar/cuboidal ductal epithelial cells with variable mucin, in all three groups of mice (Fig 2). There was no obvious difference in number or severity of lesions between the three groups of mice. Ductal cells in PanIN lesions for all groups were positive and similar in intensity for pERK staining. In all, there were no obvious differences in early tumor development in *Ksr1*^*-/-*^ mice compared to control animals in this PDAC model.

### *Ksr1*^*-/-*^ mice still develop pancreatic ductal adenocarcinoma

**Fig 3:**
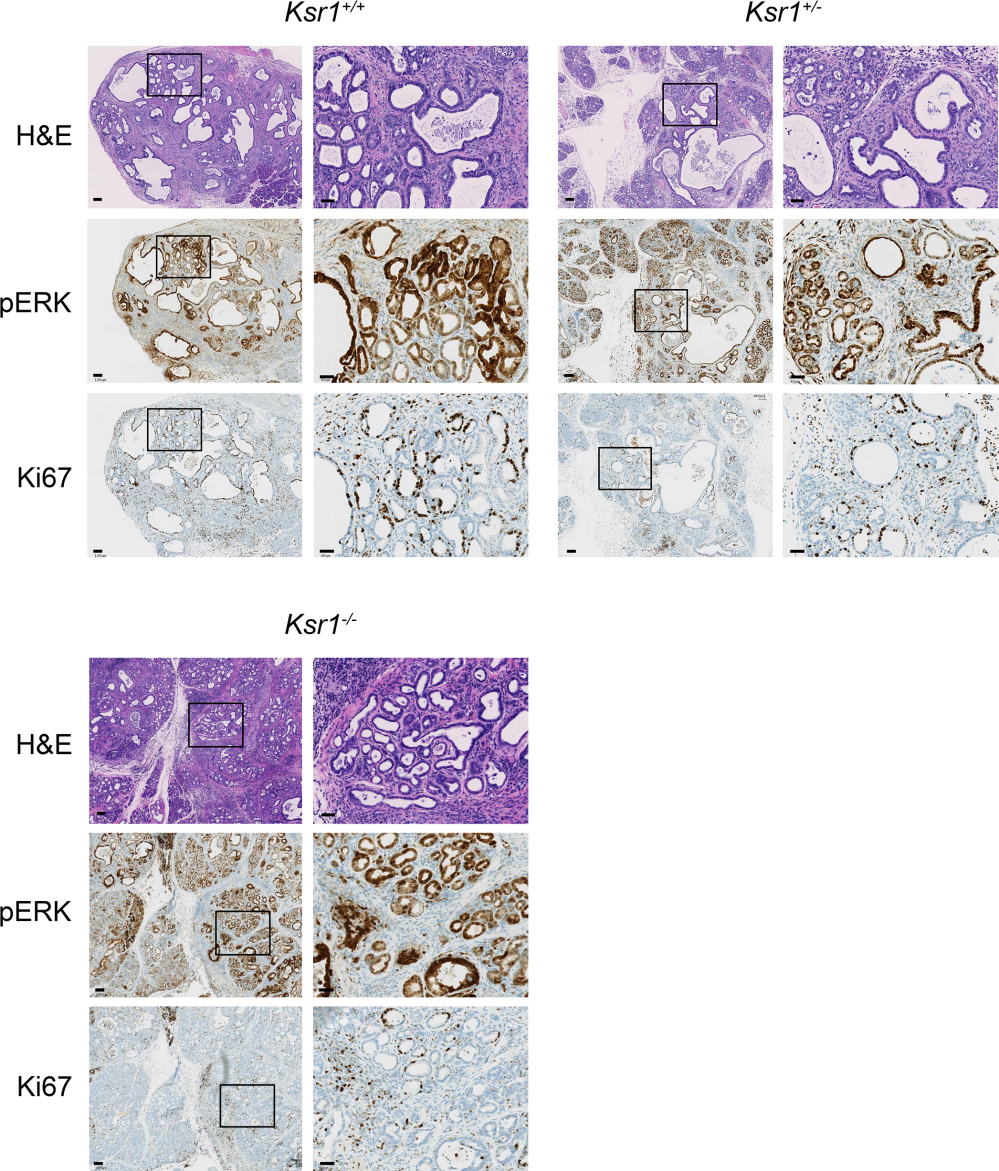
*Pdx1-Cre;LSL-Kras*^*G12D/+*^*;Trp53*^*flox/wt*^*;Ksr1*^*-/-*^ mice develop pancreatic tumors. H&E (top), pERK (middle) and Ki67 (bottom) staining of tumors harvested from *Pdx1-Cre*;*LSL*-*Kras*^*G12D/*+^*;Trp53*^*flox/wt*^*;Ksr1*^*+/+*^, *Ksr1*^*+/-*^, and *Ksr1*^*-/-*^ mice (bar = 100μm). Panels to the right show magnification of indicated region (bar = 40μm). Tumors have moderately differentiated ductal morphology that stains strongly for pERK, accompanied by stromal desmoplasia. Ki67 stains some ductal and surrounding cells.

While *Ksr1*^*-/-*^ mice were generally older before showing signs of morbidity compared to control animals, all mice eventually succumbed to pancreatic tumors. Histological analysis revealed that these tumors were ductal adenocarcinomas of similar grade to tumors examined from *Ksr1*^*+/+*^ mice, with similar patterns and intensity of pERK and Ki67 staining (Fig 3). These results demonstrate that KSR1 deficiency does not prevent progression to aggressive PDAC in a KRAS-driven model with heterozygous loss of p53.

To test the possibility that a different cancer genetic signature emerged in the absence of KSR1, we compared gene expression by RNA sequencing (RNASeq). RNASeq showed that the samples were very heterogeneous with no common or distinct molecular signature in *Ksr1*^*-/-*^ tumors compared to tumors from *Ksr1*^*+/+*^ and *Ksr1*^*+/-*^ mice (S2A Fig). A subset of the *Ksr1*^*-/-*^ and *Ksr1*^*+/-*^ samples that did cluster together (2/9 *Ksr1*^*-/-*^ and 2/7 *Ksr1*^*+/-*^ tumors) yielded some statistically significant differences, including downregulation of glucagon and insulin in the *Ksr1*^*-/-*^ samples (S2B Fig). Therefore, expression analysis did not support a distinct genetic signature in *Ksr1*^*-/-*^ compared to *Ksr1*^*+/-*^ and *Ksr1*^*+/+*^ tumors. We specifically did not observe an upregulation in KSR2 message in *Ksr1*^*-/-*^ tumors. We further confirmed that there was not a relative increase in KSR2 with real-time quantitative PCR (RT-qPCR) using the same RNA samples (S3 Fig).

### *Ksr1*^*-/-*^ animals are protected from rectal prolapse

We noted that *Ksr1*^*+/+*^ and *Ksr1*^*+/-*^ on the PDAC background developed rectal prolapse at a relatively high frequency. 4/22 *Ksr1*^*+/+*^ and 7/17 *Ksr1*^*+/-*^ mice developed rectal prolapse and were sacrificed for that reason. Interestingly, none of the *Ksr1*^*-/-*^ mice (0/19) on the PDAC background developed rectal prolapse. This did not appear to correlate with tumor burden as the size or extent of tumor was not correlated with the presence or absence of rectal prolapse. As noted in S1 Table, more female *Ksr1*^*+/-*^ mice (6/12) were sacrificed due to rectal prolapse (compared to 1/5 males), while the trend was reversed for *Ksr1*^*+/+*^ mice (1/13 females compared to 3/9 males); however, it should be stressed that our sample size is too small to draw definite conclusions based on gender. Importantly, exclusion of all animals that developed rectal prolapse from our survival curves did not change the conclusion that *Ksr1* deficient animals had a modest increase in survival compared to the *Ksr1* wild-type and heterozygous animals.

## Discussion

Given the frequency with which RAS is mutated in cancer, much effort has been focused on targeting RAS signaling for therapeutic benefit. KSR is a particularly attractive target because it is known to positively regulate MAPK signaling in the context of constitutively active RAS [1–3, 13–16, 23]. It also does not appear to be essential in normal cells, and so its inhibition might be expected to synergize with compounds that target other components of the MAP kinase signaling pathway without increasing toxicity. Indeed, one group has already reported a KSR-specific inhibitor that can synergize with the MEK inhibitor trametinib to decrease viability of RAS mutant cell lines [24]. Previously, we showed that KSR1 deficiency attenuated both the number and the growth of tumors that developed using the MMTV breast cancer model [11]. Another group used antisense oligonucleotides to demonstrate that KSR1 knockdown could reduce growth of KRAS-dependent human pancreatic carcinoma xenografts in nude mice [16].

We therefore wanted to determine the effect of KSR1 deletion in a well-characterized mouse model of KRAS-driven pancreatic cancer. We found that the loss of KSR1 resulted in a modest, but statistically significant decrease in morbidity; however, all mice eventually succumbed to pancreatic tumors. This is somewhat surprising in light of previous work using antisense oligonucleotides [16], but our study is the first to test the role of KSR1 using this genetically engineered mouse model of PDAC. It is possible that the loss of KSR1 in the stromal or immune compartment could have compensated for the loss of KSR1 in tumor cells, or that KSR1 deletion caused developmental changes which circumvented KSR1 dependence for tumors in this particular model. It is also possible that this PDAC model is so robust that it masks a role for KSR1 which might be seen in other contexts of aberrant RAS signaling. Of note, our study incorporated KSR1 depletion in all tissues, while Xing *et al*. [16] used antisense oligonucleotides. Since the efficacy of KSR1 knockdown with antisense oligonucleotides correlates with cellular uptake and not with plasma levels [25], the effect of antisense oligonucleotides may have been greater in certain cells, including tumor cells, compared with other tissues, such as the immune compartment. Future studies using a KSR1 conditional knockout could help resolve some of these discrepancies. Notably, there may be important clinical distinctions between KSR1 knockdown, altering its conformation, and complete KSR1 deficiency, and so our results do not necessarily contradict efforts to target KSR1 for therapeutic benefit.

We can conclude that in this specific mouse model of pancreatic cancer, KSR1 signaling was not required for tumorigenesis. However, this does not rule out a role for KSR in other settings, especially in light of previous reports suggesting it is a positive regulator of RAS signaling [1–3, 11, 23, 26, 27]. In a study of human breast cancer where RAS mutations are rare, high KSR1 levels were shown to correlate with overall survival, and mechanistic studies suggested that this is due to KSR1 stabilization of BRCA1 [28]. KSR may therefore have functions outside of MAPK modulation that could impact cell growth and proliferation.

Of note, in our model we used a p53 deleted allele, while most genetically engineered PDAC mouse models use the p53 R172H hypomorph [29–33]. A previous study directly comparing heterozygous deletion of p53 to heterozygous expression of p53 R172H in the same PDAC model showed no difference in tumor latency or survival. However, 13/20 of hypomorphic p53 mutant mice had liver metastases compared to 0/20 of mice with the heterozygous p53 deletion [34]. Similarly, we rarely observed liver metastases in our mice, and it is therefore possible that using the deleted p53 allele might have masked a biological role of KSR1 in promoting p53-driven metastases.

One hypothesis is that KSR2 could be compensating for the absence of KSR1. We did not, however, detect KSR2 expression in the pancreas nor did we see it upregulated in the RNASeq analysis performed on *Ksr1*^*-/-*^ mouse tumors. We further confirmed with RT-qPCR that relative KSR2 expression did not increase in *Ksr1*^*-/-*^ compared to *Ksr1*^*+/+*^ tumor samples, and so a compensatory role for KSR2 is less likely. With RNASeq, we were also unable to detect a difference in genetic signature based on KSR1 genotype. While this could be consistent with the absence of a strong phenotypical difference between *Ksr1*^*-/-*^ and *Ksr1*^*+/+*^ mice, it may also reflect the heterogeneity of the tumors analyzed. All these caveats are important to keep in mind for future studies of KSR.

One unexpected finding that arose during the course of this study was a high frequency of rectal prolapse that occurred in the *Ksr1*^*+/+*^ and *Ksr1*^*+/-*^ cohorts (4/22 and 7/17 respectively). Because of ethical concerns, we were forced to sacrifice these mice at the time we noticed the prolapse. Prolapse did not correlate with the presence of gross tumor at sacrifice, nor with the size or extent of tumor. Importantly, removal of all animals with prolapse did not have any significant effect on the overall survival statistics. We do not know why *Ksr1*^*-/-*^ mice were protected from the development of rectal prolapse, nor why its incidence has not been documented in other reports. One previous study did mention rectal prolapse in passing as a reason for censorship in survival analysis of *Pdx1-Cre;LSL-Kras*^*G12D/+*^*;Trp53*^*LSL-R172H/wt*^ mice [35]. It is possible that in our cohort, PDX1-Cre is expressed in cells in the intestine or that the absence of KSR1 in immune cells is causing a defect in the intestinal barrier through perhaps a change in the microbiome. Preliminary histology from a mouse with prolapse showed a mild to moderate enteritis in the small bowel with an inflammatory infiltrate and apoptotic cells throughout the intestinal crypts, as well as a mild increased inflammatory infiltrate in the colon. Our results suggest a protective effect in *Ksr1*^*-/-*^ mice that would be interesting to investigate in future studies.

Overall, the trend toward decreased morbidity with *Ksr1*^*-/-*^ mice that we observed was modest and all mice eventually developed invasive ductal adenocarcinoma. Even though our study does not point to an essential function for KSR1 in KRAS-driven pancreatic cancer with heterozygous loss of p53, we cannot rule out a supporting role for KSR1, or a function in other contexts, and further work will be needed to dissect the complexities of KSR signaling.

## Supporting information

S1 Table Characteristics of mice in survival cohort. Note that 7/17 *Ksr1*^*+/-*^ and 4/22 *Ksr1*^*+/+*^ mice had to be sacrificed due to the development of rectal prolapse.
(DOCX)

S1 Fig Kaplan-Meier curves for *Pdx1-Cre*;*LSL*-*Kras*^*G12D/*+^*;Trp53*^*flox/wt*^*;Ksr1*^*-/-*^ mice versus *Pdx1-Cre*;*LSL*-*Kras*^*G12D/*+^*;Trp53*^*flox/wt*^*;Ksr1*^*+/-*^ and *Ksr1*^*+/+*^ mice according to gender. While there was a trend toward *Ksr1*^*-/-*^ females sacrificed at an older age, given the sample size the difference is not statistically significant (p = 0.0683). There were also no other statistically significant differences between the different gender cohorts.
(TIF)

S2 Fig mRNA expression in tumors harvested from *Ksr1* cohorts do not cluster by genotype. A. Unsupervised clustering of mRNA expression across the entire transcriptome. *Ksr1* genotypes are indicated as KO (knockout), HET (heterozygous) and WT (wild-type). B. Two *Ksr1*^*-/-*^ and two *Ksr1*^*+/-*^ tumor samples were selected that each clustered based on whole transcriptome analysis. Depicted here are the top 100 candidates differentially expressed between the two genotypes. Four genes downregulated in *Ksr1*^*-/-*^ samples are listed with their respective p values (a complete list is included in S1 File).
(TIF)

S3 Fig Relative KSR2 expression does not increase in *Ksr1*^*-/-*^ compared to *Ksr1*^*+/+*^ tumor samples. RT-qPCR was performed on the same tumor RNA samples used for RNASeq. ΔCT values were calculated by subtracting KSR2 CT values from CT values for the reference gene, EEF2; accordingly, higher ΔCT values correspond to lower KSR2 expression. Each tumor sample is plotted as an individual point with error bars representing the standard deviation of the mean ΔCT for three technical replicates. Mean ΔCT values were not significantly different between 3 *Ksr1*^*+/+*^, 5 *Ksr1*^*+/-*^, and 5 *Ksr1*^*-/-*^ tumor samples (difference in ΔCT between *Ksr1*^*+/+*^ and *Ksr1*^*-/-*^: -0.11, 95% confidence interval [CI] -2.92 to 2.71; between *Ksr1*^*+/-*^ and *Ksr1*^*-/-*^: -0.52, 95% CI -2.96 to 1.92; between *Ksr1*^*+/+*^ and *Ksr1*^*+/-*^: 0.41, 95% CI -2.40 to 3.23). RNA extracted from mouse colon, lung and ovary was used for positive controls.
(TIF)

S1 File Top 100 genes differentially expressed between the two *Ksr1*^*-/-*^ and two *Ksr1*^*+/-*^ tumor samples. (XLSX)
