# Supplementary material for: Homozygous KSR1 deletion attenuates morbidity but does not prevent tumor development in a mouse model of RAS-driven pancreatic cancer
Source: PLoS One. 2018 Mar 29;13(3):e0194998. doi: 10.1371/journal.pone.0194998 (PMC5875795; doi:10.1371/journal.pone.0194998)
Supplement: S1 Table — Note that 7/17 Ksr1+/- and 4/22 Ksr1+/+ mice had to be sacrificed due to the development of rectal prolapse. (DOCX) [file pone.0194998.s001.docx]

| ***Ksr1* genotype** | **age at sacrifice/death** | **sex** | **notes** |
| --- | --- | --- | --- |
| *Ksr1^-/-^* | 90 | M |  |
| *Ksr1^-/-^* | 122 | F |  |
| *Ksr1^-/-^* | 140 | F | sac’d for dystocia and uterine prolapse, censored |
| *Ksr1^-/-^* | 144 | M | sac’d for slight rectal abnormality, censored |
| *Ksr1^-/-^* | 150 | M |  |
| *Ksr1^-/-^* | 158 | M | sac’d for growth on cheek, censored |
| *Ksr1^-/-^* | 163 | M |  |
| *Ksr1^-/-^* | 178 | M |  |
| *Ksr1^-/-^* | 181 | M |  |
| *Ksr1^-/-^* | 185 | M |  |
| *Ksr1^-/-^* | 187 | M |  |
| *Ksr1^-/-^* | 191 | M |  |
| *Ksr1^-/-^* | 209 | M |  |
| *Ksr1^-/-^* | 217 | F |  |
| *Ksr1^-/-^* | 225 | F |  |
| *Ksr1^-/-^* | 253 | M |  |
| *Ksr1^-/-^* | 280 | M |  |
| *Ksr1^-/-^* | 313 | F |  |
| *Ksr1^-/-^* | 396 | F |  |
| *Ksr1+/-* | 99 | M |  |
| *Ksr1+/-* | 113 | F |  |
| *Ksr1+/-* | 116 | F | runt, not growing, censored |
| *Ksr1+/-* | 120 | F | sac’d for rectal prolapse, censored |
| *Ksr1+/-* | 122 | F | sac’d for rectal prolapse, censored |
| *Ksr1+/-* | 122 | F |  |
| *Ksr1+/-* | 130 | M |  |
| *Ksr1+/-* | 138 | F | sac’d for rectal prolapse, censored |
| *Ksr1+/-* | 143 | F |  |
| *Ksr1+/-* | 146 | M | sac’d for rectal prolapse, censored |
| *Ksr1+/-* | 148 | M |  |
| *Ksr1+/-* | 152 | F |  |
| *Ksr1+/-* | 159 | F |  |
| *Ksr1+/-* | 164 | M |  |
| *Ksr1+/-* | 172 | F | sac’d for rectal prolapse, censored |
| *Ksr1+/-* | 172 | F | sac’d for rectal prolapse, censored |
| *Ksr1+/-* | 175 | F | sac’d for rectal prolapse, censored |
| *Ksr1+/+* | 49 | F | runt, losing weight, censored |
| *Ksr1+/+* | 89 | M | sac’d for rectal prolapse, censored |
| *Ksr1+/+* | 106 | M |  |
| *Ksr1+/+* | 110 | F |  |
| *Ksr1+/+* | 111 | F | sac’d for ulcerated vaginal warts, censored |
| *Ksr1+/+* | 114 | F |  |
| *Ksr1+/+* | 116 | M | censored |
| *Ksr1+/+* | 116 | F |  |
| *Ksr1+/+* | 122 | F | sac’d for rectal prolapse, censored |
| *Ksr1+/+* | 128 | F |  |
| *Ksr1+/+* | 140 | M | sac’d for rectal prolapse, censored |
| *Ksr1+/+* | 144 | F |  |
| *Ksr1+/+* | 144 | M | sac’d for tumor on paw, censored |
| *Ksr1+/+* | 148 | M | sac’d for rectal prolapse, censored |
| *Ksr1+/+* | 158 | M |  |
| *Ksr1+/+* | 160 | F |  |
| *Ksr1+/+* | 185 | F |  |
| *Ksr1+/+* | 197 | F | sac’d for possible papilloma, censored |
| *Ksr1+/+* | 197 | M | censored |
| *Ksr1+/+* | 206 | M |  |
| *Ksr1+/+* | 209 | F |  |
| *Ksr1+/+* | 243 | F |  |
