# Supplementary figures and images for: Homozygous KSR1 deletion attenuates morbidity but does not prevent tumor development in a mouse model of RAS-driven pancreatic cancer
Source: PLoS One. 2018 Mar 29;13(3):e0194998. doi: 10.1371/journal.pone.0194998 (PMC5875795; doi:10.1371/journal.pone.0194998)

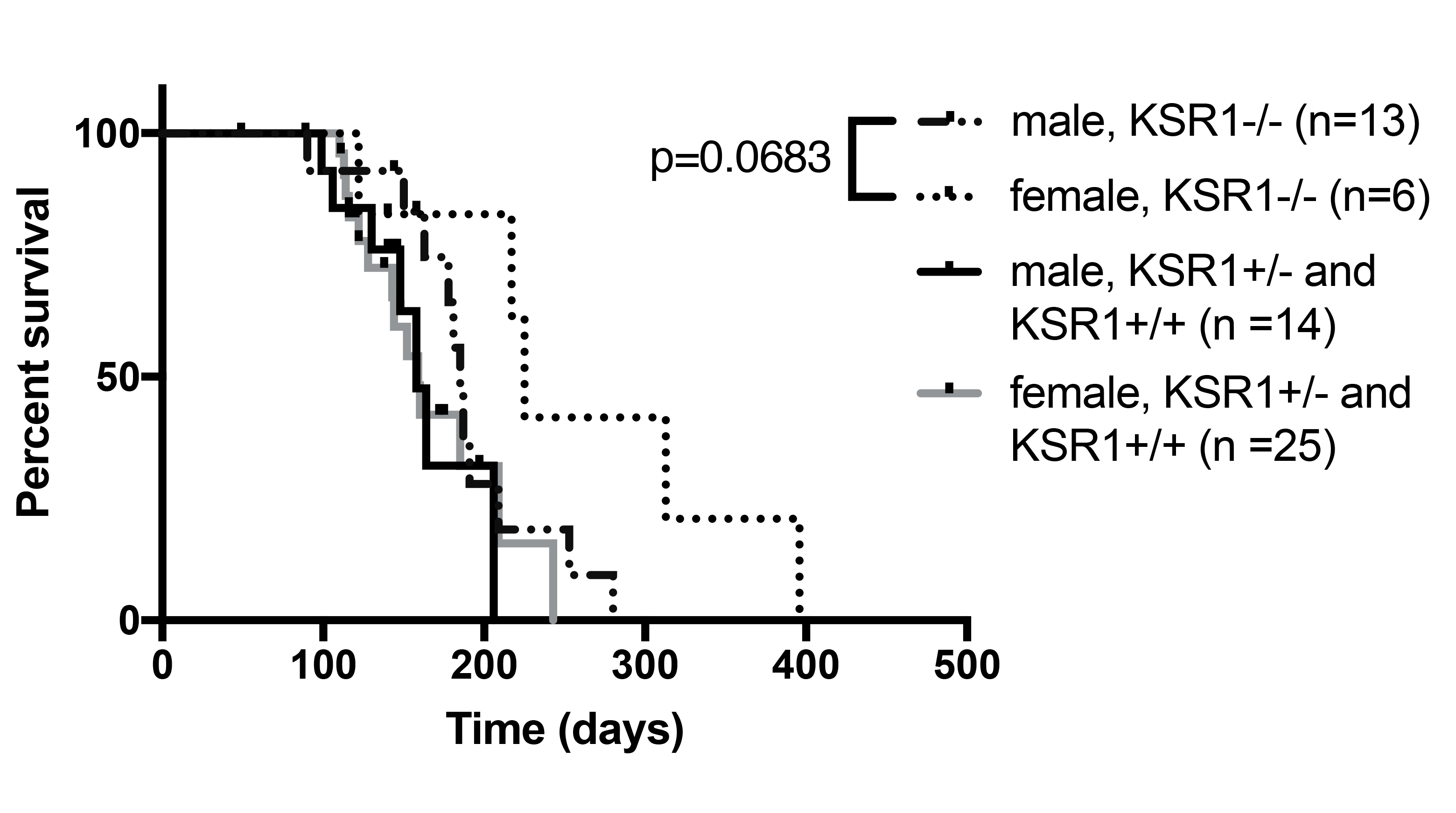

Supplement: S1 Fig — While there was a trend toward Ksr1-/- females sacrificed at an older age, given the sample size the difference is not statistically significant (p = 0.0683). There were also no other statistically significant differences between the different gender cohorts. (TIF) [file pone.0194998.s002.tif]

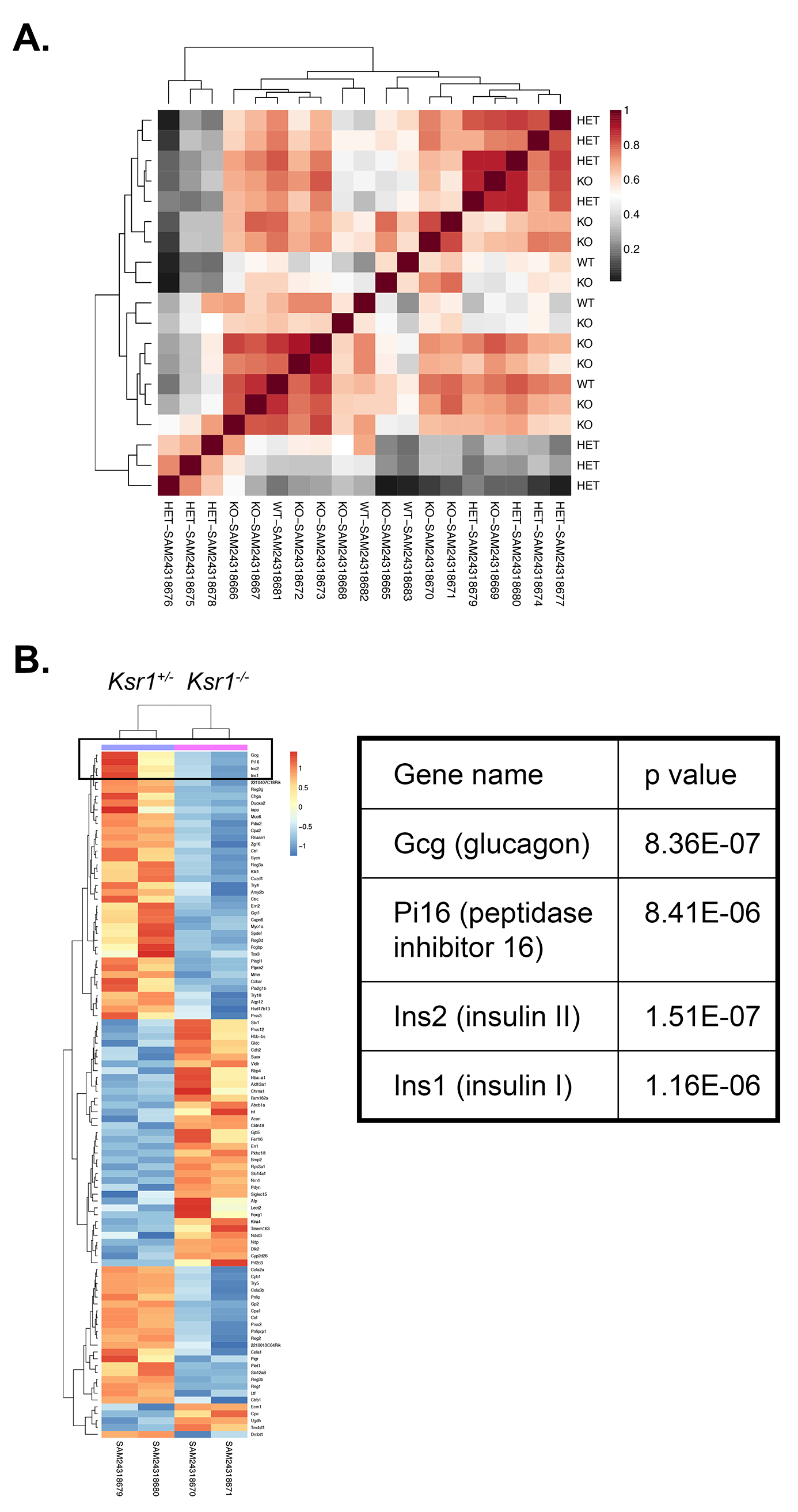

Supplement: S2 Fig — A. Unsupervised clustering of mRNA expression across the entire transcriptome. Ksr1 genotypes are indicated as KO (knockout), HET (heterozygous) and WT (wild-type). B. Two Ksr1-/- and two Ksr1+/- tumor samples were selected that each clustered based on whole transcriptome analysis. Depicted here are the top 100 candidates differentially expressed between the two genotypes. Four genes downregulated in Ksr1-/- samples are listed with their respective p values (a complete list is included in S1 File). (TIF) [file pone.0194998.s003.tif]

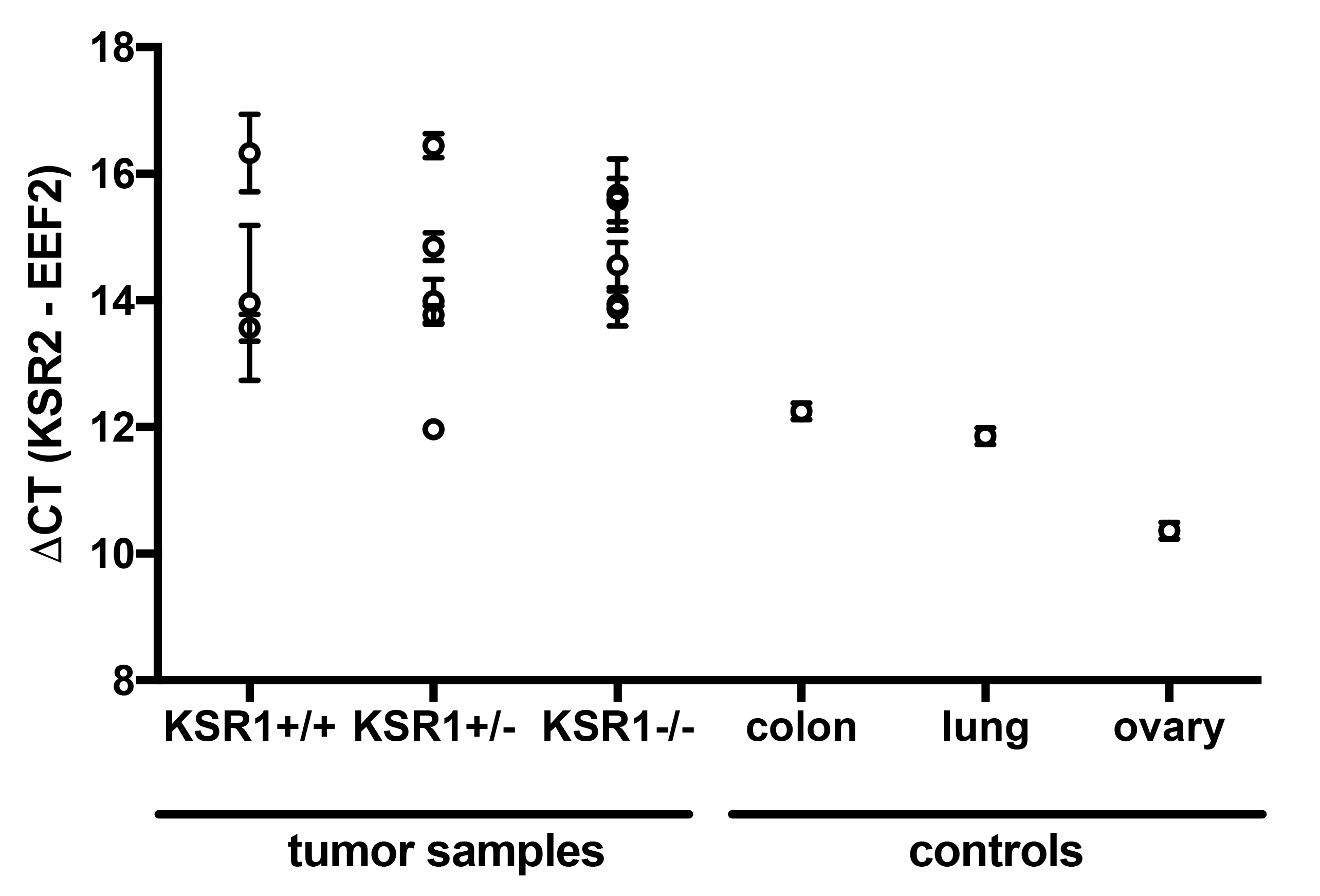

Supplement: S3 Fig — RT-qPCR was performed on the same tumor RNA samples used for RNASeq. ΔCT values were calculated by subtracting KSR2 CT values from CT values for the reference gene, EEF2; accordingly, higher ΔCT values correspond to lower KSR2 expression. Each tumor sample is plotted as an individual point with error bars representing the standard deviation of the mean ΔCT for three technical replicates. Mean ΔCT values were not significantly different between 3 Ksr1+/+, 5 Ksr1+/-, and 5 Ksr1-/- tumor samples (difference in ΔCT between Ksr1+/+ and Ksr1-/-: -0.11, 95% confidence interval [CI] -2.92 to 2.71; between Ksr1+/- and Ksr1-/-: -0.52, 95% CI -2.96 to 1.92; between Ksr1+/+ and Ksr1+/-: 0.41, 95% CI -2.40 to 3.23). RNA extracted from mouse colon, lung and ovary was used for positive controls. (TIF) [file pone.0194998.s004.tif]
